# Contextual factors influencing the implementation of a new midwife education programme in India: a qualitative study

**DOI:** 10.1186/s12909-022-03814-9

**Published:** 2022-11-04

**Authors:** Kerstin Erlandsson, Paridhi Jha, Bharati Sharma, Malin Bogren

**Affiliations:** 1 School of Health and Welfare, Dalarna University, Falun, Sweden; 2 Department for Women’s and Children’s health, Karolinska Institutet, Solna, Sweden; 3 Foundation for Research in Health Systems, Bangalore, Karnataka, India; 4 Indian Institute of Public Health Gandhinagar, Gujarat, India; 5 Institute of Health and Care Sciences, Sahlgrenska Academy, University of Gothenburg, Gothenburg, Sweden

**Keywords:** Context, Implementation science, Qualitative study, Midwifery education, Nurse-Midwife, Midwife educator, Clinical practice sites, South-East Asia

## Abstract

The Indian Government has committed to educate 90,000 midwives in accordance with international norms. This goal is critical as midwives provide evidence-based, high-quality midwifery care. There is a need to explore the contextual factors influencing this new midwifery education programme. Hence, the *aim* of this study is to explore contextual factors influencing the implementation of the national midwifery education programme for midwifery educators and the future Nurse Practitioners in Midwifery (NPMs) in India. A qualitative research design was used, with data collected through focus group discussions (*n* = 8) with a total of 27 participants representing seven national and international organisations supporting the Indian Government in its midwifery initiative. Transcribed interviews were analysed using content analysis. This study on contextual factors influencing the implementation of the new midwifery education programme in India showed that organisational and administrative processes are complex and the development of midwifery educators and nurse practitioners in midwifery needs to be fast tracked. The education of educators and future midwives in India, and elsewhere in similar settings, could benefit from efforts to simplify the organisational and administration processes and, in parallel, mobilize innovative teaching and learning approaches to bridge theory and practice.

## Introduction

A well-educated and competent midwife workforce is critical to delivering high-quality midwifery services. The countries of the South-East Asia region, and India in particular, have challenges producing adequate numbers of well-educated midwives [1]. If the Sustainable Development Goals, Agenda 2030 and specifically goal three on health, are to be achieved [2, 3], accelerated investments in high-quality education and training of midwives are needed [4].

Existing research recognizes that the length and structure of midwifery education programmes vary in and between countries in the South-East Asia region [1]. According to the International Confederation of Midwives (ICM), there are two paths to becoming a professional midwife: a three-year direct-entry midwifery education programme, and a 1.5-year post-nursing programme [5]. Both programmes should comprise a minimum of 40% theory and 50% practice [6], including at minimum 40 vaginal childbirths [7].

Midwifery in India has historically been merged with general nursing programmes [8]. To date, midwifery education is integrated into three basic professional programmes: a two-year Auxiliary Nurse Midwives certificate programme at the secondary level, a three-and-a-half-year diploma in General Nursing and Midwifery, and a four-year Bachelor’s degree in Nursing, with possibilities for a two-year Master’s programme in Nursing, Public or Reproductive Health [8]. None of these educational paths leads to midwives educated in accordance with ICM and WHO’s global norms [7, 9]. To meet such norms [10], the Indian Government has made a decision to educate 90,000 professional midwives with the focus on setting up both a midwifery education programme and midwifery-led care units (MLCUs) at public health facilities, where graduated Nurse Practitioners in Midwifery (NPMs) will be deployed [11].

It is crucial to understand the context within which an education programme is implemented. Context in implementation research includes internal or external barriers or facilitators for an intervention, that may affect its implementation [12]. Hence, understanding context is vital – including identifying what the contextual factors are and how they influence the implementation of evidence-based interventions e.g., the midwifery education programme in India. As part of an implementation project aimed at improving the health of mothers and new-borns [13], the aim of this study is to explore contextual factors influencing the implementation of the education programme of midwifery educators and the future NPMs in India. Note that the terms “midwife” and “NPM” will be used interchangeably in this study.

## Methods

### Design

A qualitative research design was used [14], and data was collected through focus group discussions (FGDs) [15] with staff representing national and international organisations across India involved in setting up midwifery education programmes. The ethical approval for the study was granted by the Institutional Review Board of the Foundation of Research in Health Systems (IORG0007693). All methods were performed in accordance with the relevant guidelines and regulations in the Belmont report [16].

### Setting

The Guidelines on Midwifery Services in India [11] aim to improve the quality of care and to ensure respectful care for women and new-borns by introducing a new cadre of midwives at state and district level. A district is an administrative division of an Indian state. As noted above, these midwives will be called Nurse Practitioners in Midwifery (NPMs). In consultation with ICM, two curricula were developed, one an 18-month curriculum leading to a post-graduate diploma for educators within the NPM programme, and one an 18-month post-basic education in midwifery, which will lead to the designation ‘NPM’ for its graduates. The first phase midwifery education entails national and state-level training in which midwifery training institutes will be strengthened to educate Indian NPM educators so that they will be equipped to educate the future NPMs at the state level.

#### Education of NPM educators

The educator programme, with entry requirement at the Master’s degree level, is a six-month programme followed by 12-months of mentoring by international midwives from countries where midwifery is well established. The programme consists of three weeks of theory, with the remaining time practice-based focusing on normal-physiology birth and the midwifery model of care. Student midwifery educators who pass the examination at the end of the six months will be posted at state and national training institutes to train NPMs.

#### Education of NPMs

The NPM education programme is intended for employed nurse-midwives who are either general nurse-midwives with a diploma or nurse-midwives holding a Bachelor’s degree in nursing with an integrated midwifery programme. Candidates must have at least two years of clinical experience working in maternity care units, covering pregnancy, maternity and neonatal care. After successfully completing the 18-month residential education, the trainees graduate as NPMs.

### Study participants and data collection

All national and international organisations who were supporting the Indian Government in its midwifery initiative (*n* = 11) were invited by email in July, 2021. Participants received written information about the study, including the fact that participation was voluntary and that they had the right to withdraw at any time without explanation. A total of seven organisations agreed to participate, and the organisations themselves selected native and international staff whose work entailed supporting the Indian Government’s midwifery initiative. A variety of healthcare professions were represented by the participants, who had both programmatic and education responsibilities. The participants gave their informed consent to participate by connecting to a digital or face-to-face meeting at an agreed-upon time.

All FGDs were conducted during the period from July, 2021, to June, 2022, by three of the authors (MB, PJ, KE). Eight FGDs with 3–4 participants in each group were conducted, a total of 27 participants. The FGDs were in English and used open-ended questions related to contextual factors influencing the implementation of the midwifery initiative (education and MLCUs): “Please tell me about what factors influence the implementation of the midwifery initiative” with the follow up question “Please give examples”. “If any challenges, how are these challenges overcome?” and “What are the implementation facilitators?” This study presents content related specifically to midwifery education, a central part in setting up MLCUs in India [17]. The interviews were audio-recorded and transcribed verbatim by a professional translator. Each FGD lasted around 60 min.

### Data analysis

The transcribed interviews were analysed following principles of qualitative inductive analysis [14]. First, all transcripts were read several times to gain an understanding of the content. Next, in new readings, meaning units were identified that answered the research question: What are the contextual factors influencing the implementation of a new midwifery education programme in India? The meaning units were then compared and sorted into codes based on similar content, which were thereafter compared and clustered into subcategories and generic categories. Quotes were used to strengthen the descriptions of the results. The analysis process was completed jointly by MB and KE, with repeated discussions with PJ and BS until full agreement was reached. An example of the analysis is shown in Table 1.

**Table 1:** Example of the data analysis process from meaning unit to generic category

Meaning Unit	Code	Subcategory	Generic Category
We are part of the national task force group who will meet and discuss what challenges there are concerning the initiative, but it is required to wait patiently for this to get running. The state task force has just been formed	Task forces are formed	Guidance by the task forces at the national, state and district level is needed	Organisational and administrative processes are complex

## Results

Contextual factors identified as influencing the implementation of the national midwifery education programme of midwifery educators and the future NPMs in India were sorted into two generic categories with respective subcategories. For an overview of the results, see Table 2.

**Table 2:** Generic categories and subcategories describing contextual factors related to the education programme of midwifery educators and the future NPMs in India

Generic Category	Subcategory
Organisational and administrative processes are complex	Guidance by the task forces at the national, state and district level is needed
	Utilizing the existing nursing workforce strains the health system
	Defined selection and admission criteria exist
	Support from international experts to train midwifery educators is needed
The development of midwifery educators and NPMs needs to be fast tracked	Education institutions need to ensure high-quality education
	Curricula informed by international norms are ready to be implemented
	Theoretical learning needs to be integrated into clinical practice
	A framework for legislation and regulation needs to be in place

## Organisational and administrative processes are complex

### Guidance by the task forces at the national, state and district level is needed

The implementation of a new midwifery education programme will need guidance by the newly formed task force at the national level. Task forces are in the inception phase for state and district levels. The state-level task forces will take the form of action groups. Task forces will consist of a variety of stakeholders, e.g., the Indian Central and the State Government ministries, nursing councils at national and state levels, teaching institutes, development partners, non-governmental organisations, professional organisations, health care managers, and other experts in midwifery. The key purpose of these groups is to steer the education and clinical midwifery practice. For example, the national task force will be responsible for the implementation of all midwifery services in the country. The responsibility of the state midwifery task forces will be midwifery education and care provision in their respective states. Once in place the district-level midwifery action groups will be more action oriented compared to the national- and state-level groups.

We are part of the national task force group who will meet and discuss what challenges there are concerning the initiative, but it is required to wait patiently for this to get running. The state task force has just been formed. (FGD 7)

### Utilizing the existing nursing workforce strains the health system

A contextual factor identified as influencing the implementation of the education programme was the strain to the existing health system if the programme builds on utilizing the existing nurse-midwife workforce. Using the existing nurse-midwife workforce may e.g., drain the health system of nurses’ services and make the acute shortage of nurses in other departments an even bigger challenge for the health system. It was suggested that in parallel with the introduction of the NPM education programme, the current nurse-midwife education programme would need to be rolled out as well.

If we draw 90,000 nurse-midwives from the existing pool and train and retain them as NPMs only for maternity services, then how will the country meet the shortage of nurses? (FGD1)

A stressor identified was that the most effective and ethical way of utilizing the existing nurse-midwives would be to ensure that talented nurse-midwives already practicing midwifery care could become the educators for the new NPM programme.

### Selection and admission criteria exist

A critical contextual factor was the selection and admission criteria for becoming an NPM educator or an NPM, specifically whether they were realistic or not in light of the expected future roles. The educators who will be trained are expected to have the capacity to deliver education in both theory and practice. In addition, they will also have to function as clinical supervisors at the midwife-led care units for the future NPMs. How these selected educators would be able to handle the expected burden of this position, bringing standards into practice, was recognized as a problem. Thus, cautiousness around the choice of who was to become such an educator was considered critically important. It was also recognized, however, that the qualities that made enthusiastic, highly motivated and high performing educators were difficult to capture in selection and admission criteria.

For the selection, I feel, we should have nurse-midwives who have at least a couple of years of clinical experience in managing births. If we pick fresh nurses, who have done only five births in their lives and then we train them and take them through the programme, how will they manage? We need to pick the right candidates and place them in the right place at the right level, and then we will see results. (FGD6)

### Support from international experts to train midwifery educators is needed

Experienced international midwives from countries where midwifery care is established had been recruited to mentor and facilitate nurse-midwives in becoming midwifery educators. A critical argument for bringing international midwives to India was to introduce the midwifery philosophy, but it was still to be in line with the Indian Government’s guidelines and customized to the Indian population. These international midwives were to be instrumental as role models bringing about the shift in thinking from a medical model of care to a midwifery one. However, greater benefit would have accrued if the group of international experts had been better coordinated. One week of introduction had been provided to them; with a coordinator in place, however, the time to have cultural humility could be shortened.

…until India develops their own educators that understand midwifery, they will probably need that support because there is no role model to follow. The role models they have currently are obstetricians. So, if we are not careful, we will produce mini doctors and we will produce NPMs that are so good at following instructions but not following, you know, what the midwifery philosophy is. So, there is a danger of that happening. (FGD 2)

The suspension of the education programme during the Covid-19 pandemic, when the international midwife mentors had to return to their home countries, showed that relying on international experts has limitations. A suggestion was made to instead find and utilize those native Indian midwives, who were already educated and functioning as midwives elsewhere in India and in the world and who held at minimum a Master’s degree in midwifery. A more sustainable solution could be found by bringing back competence to India in this way.

### The development of midwifery educators and NPMs needs to be fast tracked

#### Education institutions need to ensure high-quality education

An overarching contextual factor raised was that to ensure high-quality education the institutes had to be suitable for delivering such education. Ingredients recognized as important for maintaining high-quality education were learning and teaching material, libraries, wi-fi, and simulation-based labs. A critical factor was a quality assurance system to measure progress towards standards to ensure that the students obtained the competences required before graduating. Such a system is yet to be developed.

We are planning to do some quality control of all the National Midwifery Training Institutes to understand in what condition they are. And how the training is conducted at various sites, what the practical learning is, and what monitoring was put on hold because of the pandemic. (FGD5)

The participants explained that development partners had committed to support national training institutes and the set-up of midwifery-led care units at district and medical level hospitals, which will function as clinical practice sites for students.

#### Curricula informed by international norms are ready to be implemented

The curricula and syllabi were completed and ready to be implemented across the country. The documents initially prepared with support from the ICM had gone through further development by the Indian Nursing Council. The documents were considered to be good, but it was stressed that a lot of teaching and extensive learning by the students within a short period of time would be required. The curricula were considered short on sessions for simulation-based learning. That shortcoming will, together with an overambitious content, contribute to challenges that the educators will need to meet. The curricula were judged to have a risk- and medical-model approach for labour and birth. Given that the education will be in English, it was stressed that transferring all the content to the students would be a challenge.

So, if you look at, say, any 1 section, like say for antenatal care, you will have 1 h of theory. And then you will have almost a textbook that is to be taught in that 1 h. The question here is can you do justice to the subject? So we have been struggling and saying what is the most important thing that we know from global experience? What is it that a midwife really needs to do? What are the minimum essential competencies at the end of 6 months, when they go back to their respective states, that they are expected to have to establish a midwifery-led care unit. (FGD 3)

#### Theoretical learning needs to be integrated into clinical practice

A contextual factor was that theoretical learning will need to be integrated into clinical practice sites, because theory needs to be applied in certain real-life situations e.g., the pregnancy and childbirth journey of a woman. This emphasis benefits students in their clinical learning. With acquired knowledge and skills related to respectful care they can manage normal and complicated situations, the ability to consult with the obstetrician or paediatrician at the right time, thinking critically and reflecting. In summary, what is required to take the lead in a midwifery model of care unit.

If you look at any picture of midwifery-led care units, you will see alternate birthing positions being highlighted everywhere. You will have a ball, you will have a rope, you will have a birthing chair, you will have a mat, a colourful mat. So, it’s become the default setting of what midwives do. (FGD 2)

According to the participants, to become either an educator or an NPM, exposure to the full scope of midwifery practice, at all care levels, is needed, meaning labour and childbirth cannot be the only focus. Clinical practice sites must cover competences over the full range of sexual and reproductive health and rights practices, including family planning, post-abortion care, antenatal care, birth planning, and post-natal care.

#### A framework for legislation and regulation needs to be in place

A framework for legislation and regulation was yet to be put in place at the time of the FGDs. This framework was seen as crucial to implementing the new education programme if the new cadre were to function according to international norms. The National Nursing and Midwifery Commission Bill and the scope-of-practice documents for midwifery educators and NPMs have been circulated for comments from the nurse-midwives in India. To date this commentary process has not been completed. A separate licensure for NPMs is not yet in place; there was discussion as to whether this will happen or not as the licensure for NPMs could fall under the nursing-midwife licensure. The participants emphasised that after the NPM education programme starts, it will most likely be some time before the future NPMs can function autonomously, responsible for and taking the consequences of their performance, because obstetricians have the overall responsibility for pregnancy and childbirth care. On the one hand it was unclear whether or not the Indian Government was ready to let future NPMs practice on their own, instead of under the supervision of obstetricians. Conversely, legislation and regulation allowing NPMs to practice on their own could secure the appropriate education environment to support the students learning the required skills.

Legislation and regulation could promote high standards, philosophy of care, standards for continuing training for licencing, mentoring and supervision. But such standards were currently not in place. (FGD 3)

## Discussion

In this study we identified two overarching contextual factors influencing the implementation of a new education programme in India: (i) Organisational and administrative processes are complex; and (ii) The development of midwifery educators and NPMs needs to be fast tracked. These contextual factors can be linked to the elements described by the ICM’s Professional Framework for Midwifery, 2021. As such, these elements are critical for a tailored implementation of a midwifery education programme, and such elements are required before an occupation can be considered a profession [18]. Our discussion is situated within the ICM’s Professional Framework for Midwifery [18], to understand the implementation of a new midwifery education programme in India.

A critical contextual factor was the complexity of the organisational and administrative processes. As found in this study, the task forces had been newly formed at the national level and would be supported at the state and district levels further on. A positive finding was that these groups were to steer the education and clinical midwifery initiative to ensure high quality midwifery services in the country. Situated within the ICM’s Professional Framework for Midwifery [18] the task forces can be seen as the overall steering mechanism to promote an enabling environment for the midwifery programme. Hence, these task forces have the mandate and obligation to ensure evidence-based education that will create midwifery-led care of high quality through deployment across urban and rural areas in India. Thus, when the task forces at all levels are functioning and in place for the midwifery education programme, there will be a platform for a future enabling environment in which well-educated midwives can practice safe and evidence-based care in line with Nove et al. (2021) [2]. A prerequisite for implementing the ICM’s Professional Framework for Midwifery is to have a sufficient number of nurses who can enter the midwifery programme. One main challenge identified in this study was the shortage of nurse-midwives. Already in 2014, Chhugani (7) argued against using the existing nurse-midwife workforce to become NPMs in India because it could cause a shortage of nurses’ services.

According to the ICM’s Professional Framework for Midwifery [18], the midwifery philosophy and model of care makes midwifery care unique in relation to care from other health care professionals. The reorientation from a medical model to a midwifery model of care in India [17] can explain why the midwifery education programme in this study builds on support from experienced international midwives functioning as mentors from countries where midwifery care is already established. Our findings support the appointment of international midwives in India to guide the core values of what midwives do. Research shows that having mentors work as role models contributes to the development of students’ skills, attitudes and behaviours, to their identity as midwifes, and to the trust in physiological childbirth [19]. As such, the use of mentors can overcome the complexity of implementing respectful childbirth and reduction of the medicotechnical model of care [20]; mentors contribute by introducing a midwifery model of care that works in partnership with women to protect human rights and reproductive health and rights, respecting ethnic and cultural diversity based on ethical principles of justice, equity and dignity, all together known as the midwifery philosophy and model of care [18].

With sustainability of the midwifery program in India in mind, the use of international experts may not be the best choice. Having less than a week training for cultural humility for a country so diverse, rich and complex as India is confounding. International midwives would benefit from a longer orientation to the cultural history and context of midwifery in India, and how to work in a space without mirroring neo-colonial behaviours. Hence, the potential of bringing back midwives who have left India for better pay to be the future educators could be a way to reverse the ‘brain drain’. India has faced challenges in retaining their existing nurse workforce and nurses have migrated across the world from India, including to countries where midwifery is well established e.g., Australia, Ireland, New Zealand and the United Kingdom [21]. Again, with a view to sustainability, the next phase of the implementation could be to consider the creation of effective recruitment programmes for nurses in the diaspora who have been educated as midwives according to international standards. They could return and become midwifery experts in India.

A contextual factor was that the development of midwifery educators and NPMs needs to be fast tracked. The essential competences for midwifery practice are interlinked with all the elements of the ICM’s Professional Framework [18]. Thus, it is an encouraging finding that India has developed curricula for both educators and NPMs that are ready to be implemented, along with a strategy for integrating theory into practice through the implementation of midwifery-led care units across India. There are important differences between the two general contextual factors found in this study: (i) Organisational and administrative processes are complex; and (ii) The development of midwifery educators and NPMs needs to be fast tracked. To bridge these differences, simplifying the organisational and administrative processes could fast track the development of educators and future midwives. This fast-tracking is exemplified in the work undertaken in Africa and in Asia. Education Institutions need to ensure high-quality education across the world [10]. Blended on-site and online education and mentorship programmes have proven to provide quality in education, enhancing pedagogic skills, and developing assessment strategies aligned with the learning outcomes stated in the national curricula [1, 22–26]. Learnings from evaluations conducted on nurse-midwife education in India [27, 28], from the use of web-based virtual classrooms [1, 22–26] and from the use of mentorship [29] suggest possible opportunities for India to integrate web-based education and mentorship into the midwifery programme.

A barrier found in this study was the lack of a framework for legislation and for the regulation for quality education. According to the ICM’s Professional Framework [18], every profession has its own system of regulations to hold the profession accountable while enabling their autonomy and ensuring public safety. Midwifery regulatory functions include setting the scope of midwifery practice, pre-service education standards, registration of.

new midwives, and continuing competence throughout a midwife’s career. Moreover, the management of complaints and disciplinary procedures are also included, as are setting codes of conduct and ethical standards. Hence, the regulatory systems in India, when endorsed, will have implications for education and practice [30]. This challenging contextual factor has also been identified in other countries in the South-East Asia region (3).

## Limitations

The inductive qualitative design allowed participants to speak freely in English, strengthening the credibility as everyone spoke the same language [31]. That English was the second language for some participants, and this may have limited full participation in the FGDs. A strength of this study is that the voices of various stakeholders within the midwifery initiative in India are represented with confidentiality. This meant the participants did not feel pressure to be agreeable given the support from their organisation. One limitation could be that the representatives from the organisations may have shared the position of their organisations, instead of their own, in order to remain politically objective on the contextual factors around midwifery education.

## Conclusion and clinical implications

This study on contextual factors influencing the implementation of the new midwifery education programme in India has shown that organisational and administrative processes are complex and, furthermore, that the roll out of midwifery educators and nurse practitioners in midwifery needs to be fast tracked. Taken together, these contextual factors need to be fully taken into account to produce educators and midwives for safe and respectful midwifery-led care services. The contextual factors add to the understanding of the complexify of implementing midwifery education programmes informed by international norms in a country in which the profession is new. The education of educators and future midwives in India, and elsewhere in similar settings, could benefit from efforts to simplify the organisational and administration processes and, in parallel, mobilize innovative teaching and learning approaches to bridge theory and practice.

## Data Availability

With regards to confidentiality and ethical concerns, access to raw data can be provided on request from PhD Paridhi Jha, Foundation for Research in Health Systems, Bangalore, Karnataka, India: e-mail: admin@frhsindia.org.
